# Intravitreal injection of the synthetic peptide LyeTx I b, derived from a spider toxin, into the rabbit eye is safe and prevents neovascularization in a chorio-allantoic membrane model

**DOI:** 10.1186/s40409-018-0168-5

**Published:** 2018-11-21

**Authors:** Flavia Rodrigues da Silva, Mayara Rodrigues Brandão de Paiva, Lays Fernanda Nunes Dourado, Rummenigge Oliveira Silva, Carolina Nunes da Silva, Bruna Lopes da Costa, Cibele Rodrigues Toledo, Maria Elena de Lima, Armando da Silva-Cunha

**Affiliations:** 1 Faculdade de Farmácia, Universidade Federal de Minas Gerais, Av Antônio Carlos, 6627, 2nd Floor, Room 2031, Pampulha, Belo Horizonte, Minas Gerais 31270-901 Brazil; 2 Departamento de Bioquímica e Imunologia, Instituto de Ciências Biológicas, Universidade Federal de Minas Gerais, Belo Horizonte, 31270-901 Brazil

**Keywords:** *Lycosa eritrognatha.* LyeTx I b. intravitreal injection. Retinal diseases. Toxicity. Retinal neovascularization

## Abstract

**Background:**

The great diversity of molecules found in spider venoms include amino acids, polyamines, proteins and peptides, among others. Some of these compounds can interact with different neuronal receptors and ion channels including those present in the ocular system. To study potential toxicity and safety of intravitreal injection in rabbits of LyeTx I b, a synthetic peptide derived from the toxin LyeTx I found in venom from the spider *Lycosa eritrognatha* and to evaluate the angiogenic activity on a CAM model.

**Methods:**

ARPE-19 cells were treated with LyeTx I b (0.36; 0.54; 0.72; 2.89; 4.34 or 9.06 μM). In this study, New Zealand rabbits were used. LyeTx I b (2.89 μM) labeled with FITC dissolved in PBS, or only PBS, were injected into vitreous humor. Electroretinogram (ERG) was recorded 1 day before injection and at 7, 14 and 28 days post-injection. Clinical examination of the retina was conducted through tonometer and eye fundus after ERG. Eyes were enucleated and retinas were prepared for histology in order to assess retinal structure. CAMs were exposed to LyeTx I b (0.54; 0.72; 2.17 or 2.89 μM).

**Results:**

ARPE-19 cells exposed to LyeTx I b showed cell viability at the same levels of the control. The fluorescence of LyeTx I b labeled with FITC indicated its retinal localization. Our findings indicate ERG responses from rats injected in the eye with LyeTx I b were very similar to the corresponding responses of those animals injected only with vehicle. Clinical examination found no alterations of intraocular pressure or retinal integrity. No histological damage in retinal layers was observed. CAM presented reduced neovascularization when exposed to LyeTx I b.

**Conclusions:**

Intravitreal injection of LyeTx I b is safe for use in the rabbit eye and prevents neovascularization in the CAM model, at Bevacizumab levels. These findings support intravitreal LyeTx I b as a good candidate to develop future alternative treatment for the retina in neovascularization diseases.

## Background

Diseases involving the retinal vasculature, including age-related macular degeneration (AMD), diabetic retinopathy and various posterior forms of uveitis, are important causes of blindness in both industrialized countries and developing nations [1]. Diabetic retinopathy affects approximately one-third of all persons who suffer from diabetes mellitus [2], a disease related to neovascularization [3]. Diabetic retinopathy is routinely classified by clinical severity as non-proliferative or proliferative [4]. Proliferative disease is distinguished by the presence of retinal neovascularization [1].

AMD presents choroidal neovascularization (CNV) that originates from the choroid, penetrates Bruch’s membrane and develops into the sub-retinal pigment epithelial (sub-RPE) space, with accompanying exudative changes involving fluid and hemorrhaging [5, 6]. RPE elevation and enlargement of the sub-RPE space result from fluid, hemorrhaging, or the neovascular component itself [7].

The use of anti-vascular endothelial growth factor (VEGF) treatment reduced the prevalence of blindness and visual impairment due to AMD [8]. However, the primary goals of maintenance anti-VEGF therapy are achieving control of disease activity and avoiding recurrences with minimal substantial sensory retinal impairment [8]. In this sense is very important to investigate new molecules capable of preventing neovascularization without altering sensorial layers.

Spider venoms and bioactive peptides contain diverse peptide toxins, which have attracted great attention as promising drug leads and excellent research tools in pharmacology and neurobiology [9, 10]. Wolf spiders, or tarantulas, from the genus Lycosa are very common in urban areas in the southeastern region of Brazil. Our group previously isolated, characterized and chemically synthetized a peptide denominated LyeTx I from the venom of the spider *Lycosa erythrognatha*. LyeTx I contains 25 amino-acid residues, with the primary structure as follows: IWLTALKFLGKNLGKHLAKQQLAKL-NH2, and we demonstrated by NMR studies that it forms an alpha helix when interacting with membrane [11]. This peptide shows a wide antibacterial and antifungal activity [11]. Subsequently, it was tested alone or formulated with beta-cyclodextrin in periodontal pathogens and was proposed for the treatment of periodontitis. Besides its antimicrobial activity LyeTx I was also able to inhibit the proliferation of epithelial cells (a problem in this disease) at concentrations that are non-cytotoxic to osteoblasts and erythrocytes [12, 13]. In addition, the peptide, formulated or not with cyclodextrin, was effective at eradicating the multispecies 2-day biofilm at double the MIC concentrations [13].

Aiming to minimize structure and optimize action, a peptide derived from LyeTx I, called LyeTx I b, was synthesized. In contrast to LyeTx I, the derived peptide LyeTx I b has an acetylated N-terminal and an amino acid deletion, i.e., a His residue in the sixteenth position, as structural modifications. This change evoked a 10-fold increase in bactericidal activity compared to LyeTx I [14].

It was already shown that some peptides from the spider venoms are active in the ocular systems reducing glutamate content and cell death of retinal ischemic slices [15]. However, although the antimicrobial effectiveness of LyeTx I b had been demonstrated, its possible action on the eye remains unknown. Therefore, the present work aimed to investigate the safety of intravitreal injection of LyeTx I b into rabbits’ eyes, its possibly toxicity to the retina, and also to evaluate its application to prevent neovascularization in a CAM model. This work provides strong evidence that this peptide could become a valuable tool for future studies or a new therapy to prevent retinal neovascularization.

## Materials and methods

### Materials

DMEM-F12 (1:1) medium (Gibco/Carlsbad, CA), fetal bovine serum (FBS) (Gibco/Carlsbad, CA), penicillin streptomycin, amphotericin B (PSA) (Gibco/Carlsbad, CA), PBS and trypsin-EDTA (Gibco/Carlsbad, CA). Tris-base, trichloroacetic acid (TCA) (Sigma-Aldrich /St. Louis, MO), sulforhodamine B (SRB) (Sigma-Aldrich /St. Louis, MO), acetic acid (CH_3_COOH) (Sigma-Aldrich /St. Louis, MO). Ketamine, Xilasin and Mydriacil. The injected eyes were monitored by a handheld portable tonometer (Reichert Tonopen XL/ New York, USA), ophthalmoscopy Clear View® (Optibrand, Colorado, USA), electroretinography (ERG), and histology. Peptides LyeTx I b and LyeTx I b with FITC (Fluorescein Isothiocyanate) conjugate were synthetized at GenOne Biotechnologies, at Rio de Janeiro – RJ, Brazil.

### Methods

#### ARPE-19 cell culture and cytotoxicity evaluation

ARPE-19 cells (Cellular Bank of Rio de Janeiro, Brazil) were maintained in DMEM-F12 (1:1) medium supplemented with 10% fetal bovine serum (FBS) and 1% antibiotics (PSA- penicillin, streptomycin, amphotericin-B). Cells were incubated in 5% CO_2_/95% O_2_ humidified air at 37 °C for the duration of the experiment. The cell viability assay used was the sulforhodamine B (SRB) colorimetric method for toxicity screening. The day before the experiment, cells were seeded onto 96-well plates at a concentration of 10,000 cells/well. Cell concentration was determined by the Neubauer Chamber. After the treatment with the peptide, medium was replaced and cells were fixed by adding 100 μL of 10% Trichloroacetic Acid (TCA) for 1 h at 4 °C. Next, cells were washed with H_2_O and stained with 100 μL of a 0.057% SRB solution in 1% acetic acid (HAc) for 30 min at room temperature. After staining cells were washed with 1% HAc to remove the excess of SRB and then incubated with 100 μL of 10 mM Tris base, pH 10.5 and shaken for 5 min to solubilize the protein-bound dye. Absorbance was measured at 510 nm, using an ELISA plate reader (Bio-rad, San Diego, CA, USA) at 510 nm. Three wells per dose were counted in three independent experiments. Cell viability was calculated as a percentage of the control using the software GraphPad Prism v.5.0. Furthermore, morphological changes were not observed in the cells treated with different concentrations of LyeTx I b by microscopic examination. Cells were visualized (5X) using a Zeiss microscope (Axio Imager M2, Zeiss) and images were captured with a digital camera coupled to it.

#### Animals

Female New Zealand rabbits, aged approximately three months and weighing 2 kg, were purchased from the Professor Hélio Barbosa Experimental Farm (Igarapé, Brazil). The animals remained in individual cages throughout the period of adaptation (1 week) and experimentation (28 days), in an environment with an average temperature of 25 °C, constant, and brightness varying according to sunlight. There was no restriction of water or food during the experiment**.** The study was approved by the Committee for Ethics in Animal Experimentation of the Federal University of Minas Gerais (CETEA, Belo Horizonte, Brazil, Protocol n° 298/2017). The entire experiment was conducted in accordance with the Association for Research in Vision and Ophthalmology (ARVO).

#### Intravitreal injection

Twelve female New Zealand rabbits were assigned to four groups (*n* = 3 in each group), which received LyeTx I b diluted in PBS. Before all intravitreal injections, the rabbits were anesthetized by an intramuscular combination of ketamine hydrochloride (30 mg/kg) and xylazine hydrochloride (4 mg /kg). The pupils were dilated with topical 0.5% tropicamide (Mydriacyl; Alcon, São Paulo, Brazil) and the eyes were topically anesthetized with 0.5% proxymetacaine hydrochloride (Anestalcon; Alcon, São Paulo, Brazil). The eyes were wiped with 5% povidone iodide, and intravitreal injections were performed using a 30-gauge needle attached to a tuberculin syringe inserted ∼3 mm posterior to the limbus. The needle was held in place for 5 s before withdrawal to prevent reflux from the entry site. The right eye (RE) was injected with 0.1 mL of the LyeTx I b diluted in PBS and the left eye (LE) with 0.1 mL of the suspension vehicle (PBS). Control group refers to animals whose eyes were not injected.

#### Electrophysiological recordings (ERG)

ERGs were carried out in compliance with the International Society for Clinical Electrophysiology (ISCEV) guidelines [16]. ERG was performed at baseline and at 7, 14 and 28 days after the injection. ERGs were recorded using an Espion e2 electrophysiology system and a Ganzfeld LED stimulator (ColorDome™ desktop Ganzfeld, Diagnosys LLC, Littleon, MA). All ERGs were recorded after 3 h of darkness adaptation. The pupils were dilated using one drop of 0.5% tropicamide (Mydriacyl; Alcon, São Paulo, Brazil) 15 min before ERG measurement and the animals were anesthetized by intramuscular injection (ketamine hydrochloride 30 mg/kg and xylazine hydrochloride 4.0 mg/kg) before the recording of ERG. The eyes were topically anesthetized with 0.5% proxymetacaine hydrochloride (Anestalcon; Alcon, São Paulo, Brazil) immediately before the ERG recordings. Bipolar contact lenses and an electrode were placed on both corneas with 2% *w*/*v* Carboxymethyl cellulose and a needle electrode was inserted into the back. Impedance was set to less than 5 kΩ at 25 Hz in each electrode.

The darkness-adapted (scotopic) ERG protocol was recorded according to a modified ISCEV protocol and presented in the following sequence: rod (0.01 cd.s/m^2^), combined response (3 cd.s/m^2^) and high-intensity response (10 cd.s/m^2^); with 30s inter-stimulus interval (ISI), with a duration of 4 ms.

The photopic ERG protocol consisted of an initial light adaptation phase for 10 min with background illumination of 30 cd/m^2^, after which the cone single flash response was performed with luminance flashes at 3 cd.s/m^2^, and 4 ms duration (ISI = 2 s) followed by a 30-Hz white flicker stimulus of the same luminance and duration.

#### Clinical evaluation

The intraocular pressure (IOP) was measured after electroretinography using a portable tonometer (Reichert Tonopen XL/ New York, USA). At each measurement, the eyes were locally anesthetized with a 20-uL drop of 0.5% proxymetacaine hydrochloride (Anestalcon; Alcon, São Paulo, Brazil) and the IOP was measured three times to obtain the average value. The intraocular pressure changes were observed in each group (*n* = 3) with the intraocular pressure of the control eye being subtracted from that of the test eye. The eyes were examined with indirect fundus ophthalmoscopy (Welch Allyn, USA) before and after intravitreal injection to detect possible damage such as hemorrhaging, edema and inflammation caused by LyeTx I b.

#### LyeTx I b + FITC intravitreal injection

In order to determine the localization of LyeTx I b in the eye, four female New Zealand rabbits received this peptide (2.89 μM). LyeTx I b conjugated with FITC was injected into the vitreous humor, in a lightless condition, using the same protocol as described before. After 2 h, 4 h, 6 h and 8 h one animal was euthanized using overdose of barbiturate (sodium pentobarbital at a concentration of 81 mg/kg) and the retina was removed and submitted to histologic analysis. Images were acquired from fluorescence microscope (Apotome.2, ZEISS, Germany) with a 20× objective. FITC was excited at 490 nm and emission at 526 nm.

#### Histological evaluation

After the last ERG recording on day 28, animals were sacrificed and eyes were processed for light microscopy. Immediately after sacrifice, eyes were enucleated, and the posterior segment was fixed in Davidson solution (two parts 10% neutral phosphate-buffered formalin, three parts 95% ethanol, one part glacial acetic acid and three parts ultrapure water). Samples were included in paraffin and cut into 4-μm-thick sections in the sagittal plane to allow dorsal-to-ventral observation of the retina; they were stained with hematoxylin and eosin and were analyzed in unmyelinated areas under light microscopy using a microscope (Zeiss®, Model Axio Imager M2). Eyes injected with LyeTx I b were compared with vehicle-injected fellow eye of the same animal. Thickness and gross organization of each retinal layer were analyzed using the software Image J.

#### The chorio-allantoic membrane procedure

The CAM technique was performed to measure the toxicity, biocompatibility and antiangiogenic activity of LyeTx I b on 72 eggs (*n* = 12 for each group) [17]. The procedure has been found to be an acceptable alternative to in vivo tests and was performed according to [17] with minor modifications. Fertilized eggs were purchased from Rivelli (Igarapé Brazil) and placed in a rotating incubator in a humidified atmosphere at 37 °C until testing on day 5. The shell above the air cell of the eggs and the inner membrane were removed using forceps and the CAM was assessed. LyeTx I b (0.7 and 2.89 μM) was applied directly onto the CAM which was then examined for 72 h by obtaining a photo with a light microscope (Leica, model DM4000B, Germany) coupled to a Leica digital CCD camera model DFC 280 (Software Leica Application Suite V 3.3.0, Germany) illumination (Leica, model DM4000B, Germany). Each concentration of LyeTx I b was tested 12 times and the experiment was repeated once. Neovascularization was measured using the software Image J. Densitometric and nonsaturated vessels were analyzed according to the number of pixels.

#### Morphological evaluation of the CAM

In order to perform the morphological evaluation, the CAM of each egg was detached and submerged for fixation in 10% buffered formalin, for 48 h, and then embedded in paraffin. Sections 5-mm-thick were then cut by using a microtome; hematoxylin and eosin staining was then performed using an optical microscopic (Zeiss®, Model Axio Imager M2).

### Data analysis

Means ± SD are shown for the number of independent experiments indicated in Figure Legends. The software GraphPad Prism™ was employed to analyze data for statistical significance determined by analysis of variance (ANOVA) testing followed by Bonferroni post-hoc multiple comparison testing for ARPE-19 cells and CAM assay experiments.

## Results

### LyeTx Ib maintains viability of ARPE-19 culture above 50%

**Fig. 1:**
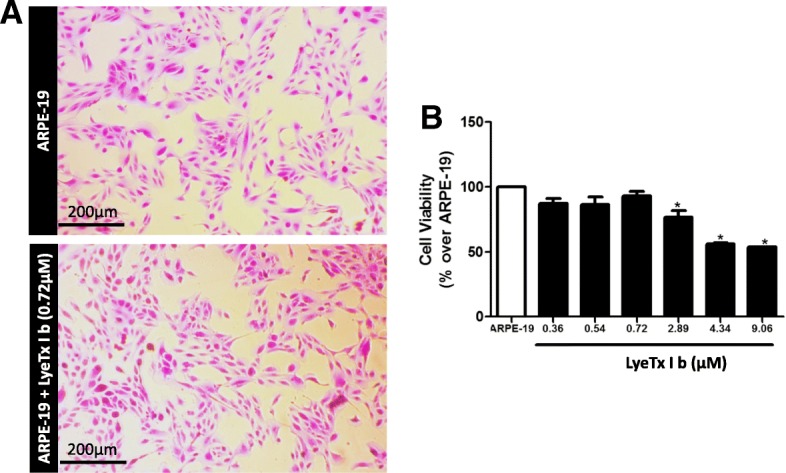
LyeTx I b does not alter the morphology of ARPE-19 cells and maintains cell viability above 50%. **a** Figure shows ARPE-19 cells in the absence or presence of LyeTx I b (2.89 μM) indicating a healthy culture. **b** Graph shows cells not exposed (ARPE-19) or exposed to LyeTx I b (0.36, 0.54, 0.72, 2.89, 4.34 or 9.06 μM). Data represent the means ± SEM of three independent experiments. * indicates significant difference as compared to ARPE-19 (*p* < 0.05). Abbreviations: SEM, standard error of the mean. Cells were visualized (5X) using a Zeiss microscope (Axio Imager M2, Zeiss) and images were captured with a digital camera coupled to it

The ARPE-19 cells are involved in many ocular inflammatory diseases that may end in loss of vision and blindness [18]. Based on a study of LyeTx I activity [11], different concentrations of LyeTx I b were tested on ARPE-19 cells: 0.36; 0.54; 0.72; 2.89; 4.34 and 9.06 μM. Our findings show that in the presence of LyeTx I b, cells morphology was not affected (Fig. 1a), indicating that the cell culture was healthy. In addition, LyeTx I b at concentrations of 2.89, 4.34 and 9.06 μM, despite promoting reductions in the number of cells, maintained respective cell viabilities of 76.89, 56.16 and 53.94% (Fig. 1b). It can be inferred that, within the range of concentrations tested, LyeTx I b does not present significant cytotoxic effects that would be able to drastically reduce cell viability, suggesting the safety of this peptide for ocular use.

### LyeTx I b intravitreal penetrates the retina of rabbits in a short time period

**Fig. 2:**
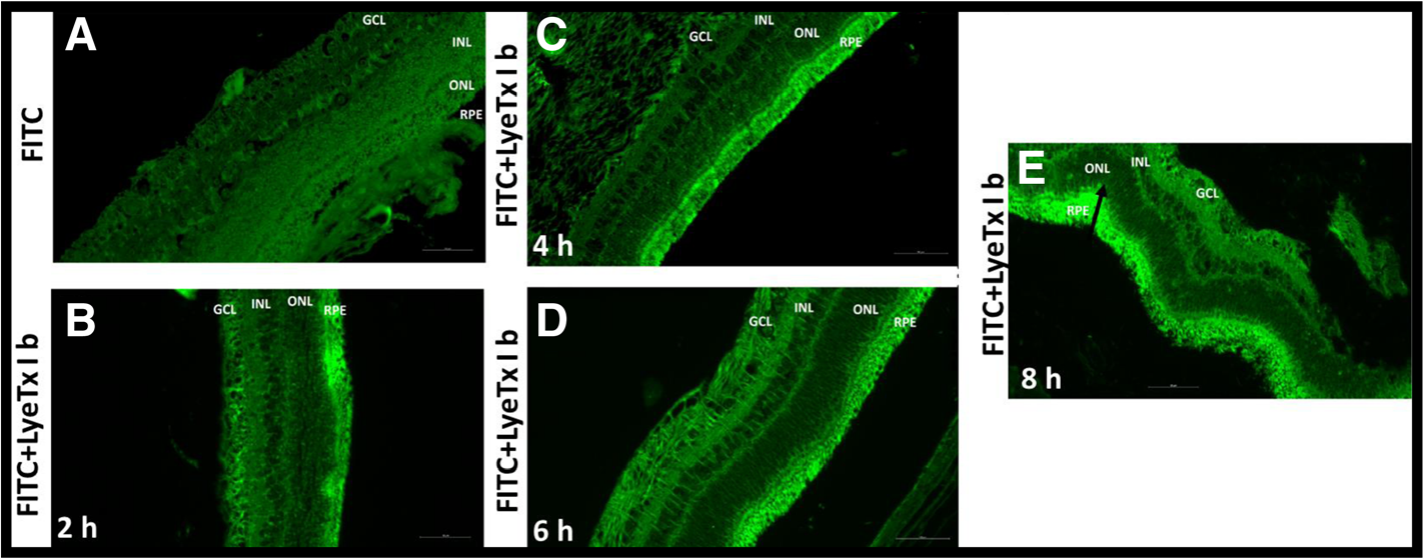
LyeTx I b – FITC intravitreal penetrates the retina. **a** The retina layers without peptide. **b** Retina layer 2 h after intravitreal injection of LyeTx I b - FITC (2.89 μM). **c** 4 h after intravitreal injection. **d** 6 h after intravitreal injection. **e** 8 h after intravitreal injection. RPE- Retinal Pigment Epithelium, ONL- Outer nuclear layer, INL- Inner nuclear layer, GCL-Ganglion cell layer. Digital images were obtained using a microscope (Apotome.2, ZEISS, Germany) equipped for epifluorescence and a standard fluorescein filter with a 20× objective. FITC was excited at 490 nm and presented emission at 526 nm

Verifying the absence of in vitro toxicity of LyeTx I b, we initiated the investigation of safety of intravitreal injection of this peptide and its affinity for the retina of rabbits. We injected LyeTx I b conjugated with FITC to certify of the presence of this peptide on retinal layers. Fluorescence promoted by FITC indicates that after intravitreal injection, LyeTx I b progressively increased its penetration with time, such that 2 h (Fig. 2b) < 4 h (Fig. 2c) < 6 h (Fig. 2d) < 8 h (Fig. 2e). The arrows indicates the increasing of the fluorescence mainly in the Retinal Pigment Epithelium (RPE).

### LyeTx I b is safe for intravitreal administration

**Fig. 3:**
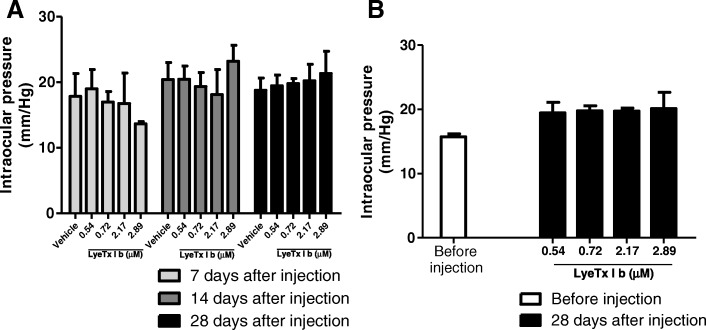
LyeTx I b intravitreal injection does not interfere with intraocular pressure: **a** Graph shows the safety of intravitreal injection of LyeTx I b (0.36, 0.54, 0.72 or 2.89 μM) 28 days after injection indicating no alterations compared to control group (Before injection). **b** Graph shows the safety of intravitreal injection of LyeTx I b (0.36, 0.54, 0.72 or 2.89 μM) 7, 14 and 28 days after injection indicating no alterations compared to vehicle. Data represent the means ± SEM of three independent experiments. The abbreviation n.s. indicates no significant difference as compared to before injection or vehicle groups (*p* > 0.05). Abbreviations: SEM, standard error of the mean

The safety of retinal application of LyeTx I b could be observed by tonometer evaluation. We observed that when LyeTx I b was injected for 7, 14 or 28 days at the concentrations 0.54; 0.72; 2.17 or 2.89 μM, the intravitreal injection did not affect intraocular pressure of the rabbits (Fig. 3a). Furthermore, we observed that LyeTx I b did not alter the intraocular pressure after the procedure (Fig. 3b).

### LyeTx I b does not compromise visual acuity

**Fig. 4:**
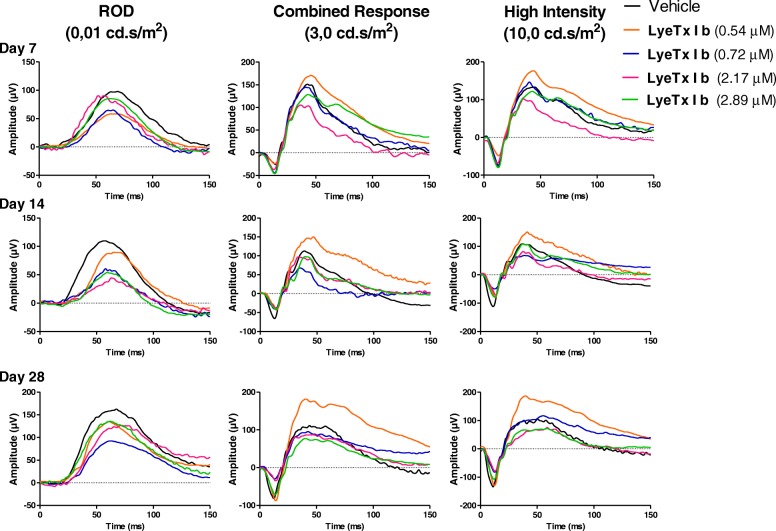
Representative ERG recordings of rabbit’s eye injected with different doses of LyeTx I b at different time points darkness-adapted (0.01, 3.0 and 10 cd.s/m^2^)

**Fig. 5:**
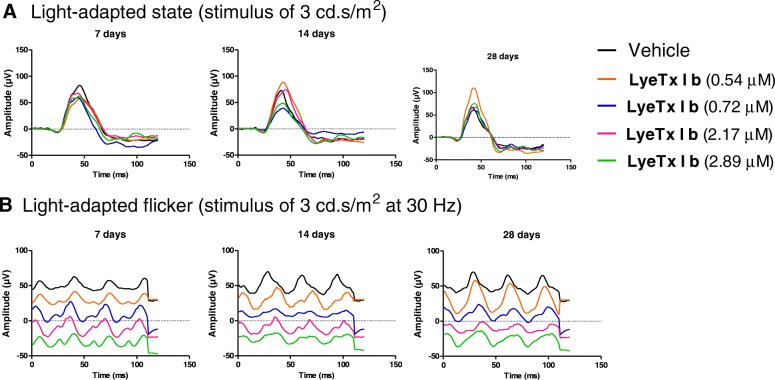
Representative ERG recordings of rabbit eyes injected with different doses of LyeTx I b at different time points (**a**) light adapted (3.0 cd.s/m^2^) (**b**) light-adapted flicker (stimulus of 3.0 cd.s/m^2^ at 30 Hz)

**Fig. 6:**
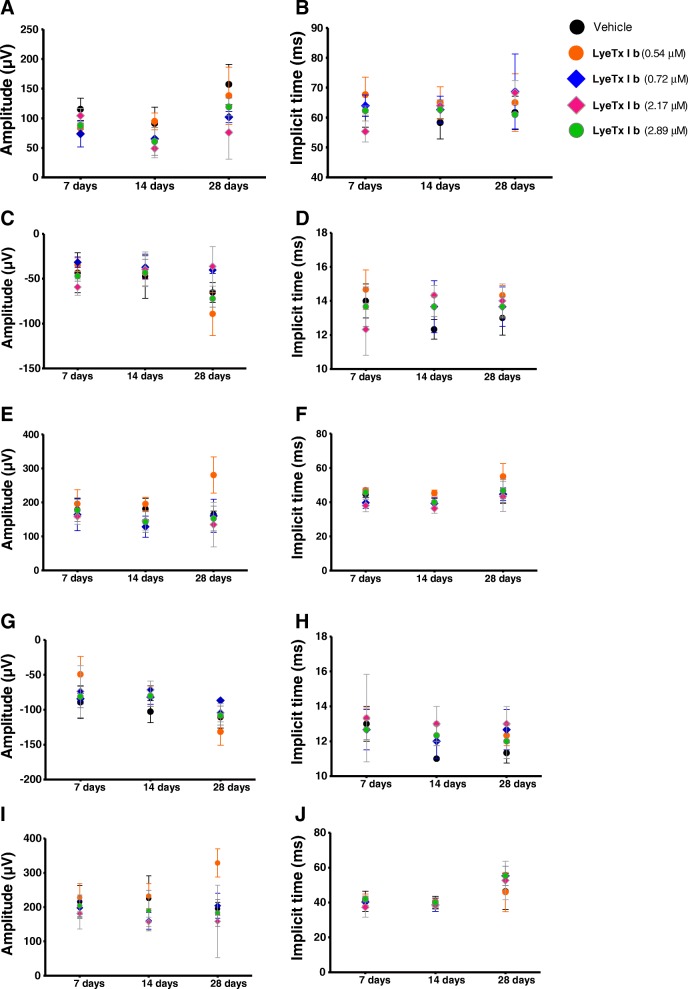
ERG darkness-adapted b-wave amplitude variation (**a**) and implicit time (**b**) in the experimental eyes with a stimulus of 0.01 cd.s/m^2^. ERG darkness-adapted a-wave amplitude variation (**c**), a-wave implicit time (**d**), a-wave amplitude variation (**e**) and b-wave implicit time (**f**) in the experimental eyes with a stimulus of 3 cd.s/m^2^. ERG darkness-adapted a-wave amplitude variation (**g**), a-wave implicit time (**h**), a-wave amplitude variation (**i**) and b-wave implicit time (**j**) in the experimental eyes with a stimulus of 10 cd.s/m^2^

**Fig. 7:**
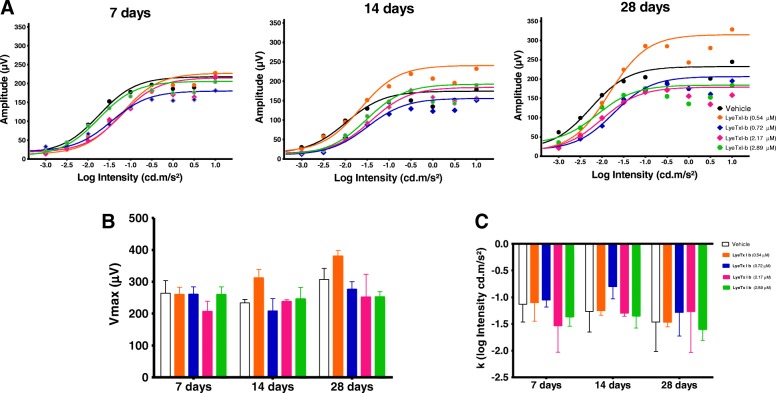
Log b-wave amplitude versus flash-intensity curves of eyes injected with different doses of LyeTx I b (**a**). Mean difference of the b-wave saturating amplitude (V_max_) (**b**). Mean difference of the log semi-saturation constant (k) of the b-wave (**c**)

Darkness and light-adapted representative ERG records obtained at 7, 14, and 28 days after the injection of intravitreal LyeTx I b at doses of 0.54, 0.72, 2.17 and 2.89 μM are shown in Figs. 4 and 5, respectively. Amplitude and implicit time are displayed in Fig 6. Our findings indicate that the group injected with LyeTx I b 0.72 μM showed a lower b-wave amplitude in the darkness-adapted rod mediated response 28 days after intravitreal injection (Fig. 5a) compared with the controls. No statistically significant differences were found between vehicle values and post-injection values on days 7, 14, and 28 at other LyeTx I b doses tested for the amplitude and the implicit wave time (which represents photoreceptor function) or b wave implicit time in the ERG response to single flash white light. We observed in the group injected with LyeTx I b at 0.54 μM an increase in their light adapted b-wave amplitude response to the single-flash white light and to the 30 Hz flickering white light compared with the vehicle 28 days after intravitreal injection. At all other injected concentrations, no differences were observed between the ERG responses of the experimental and control eyes in the light-adapted condition. LyeTx I b 0.54 μM treatment caused an increasing of b-wave amplitude in the darkness-adapted combined responses from photoreceptors and bipolar cells (Fig. 6e) and in the darkness-adapted high intensity response (Fig. 6i) compared to the vehicle-injected rabbits 28 days after intravitreal injection. Naka–Rushton parameters (*Vmax:* maximal b-wave amplitude and *k:* semi-saturation constant) for each dose of LyeTx I b and time point were obtained from b-wave amplitude versus flash intensity curves in the darkness-adapted state (Fig. 7). We did not observe differences in b-wave amplitude versus flash intensity curves in the darkness-adapted state, *Vmax* or *k.*

### Retinal vasculature is not altered after intravitreal LyeTx I b

**Fig. 8:**
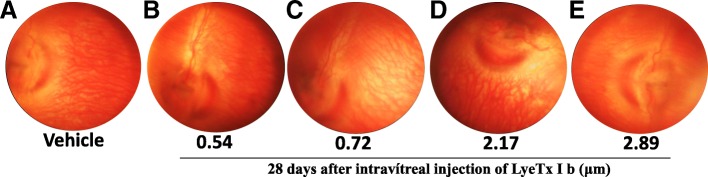
Intravitreal LyeTx I b does not damage retinal vasculature: Clinical exam is shown through Clear View images indicating no damage to retinal vasculature 28 days after intravitreal injection of (**a**) Vehicle, (**b**) LyeTx I b 0.54 μM, (**c**) LyeTx I b 0.72 μM, (**d**) LyeTx I b 2.17 μM and (**e**) LyeTx I b 2.89 μM

Eye fundus was performed after intravitreal injections of LyeTx I b at the following concentrations: 0.54; 0.72; 2.17 and 2.89 μM at 7, 14 and 28 days. We found that LyeTx I b did not alter retinal vasculature at 7 or 14 days (data not shown) and for a long period (28 days) was safe at all concentrations studied compared to the control (Fig. 8).

### LyeTx I b does not alter retinal morphology integrity

**Fig. 9:**
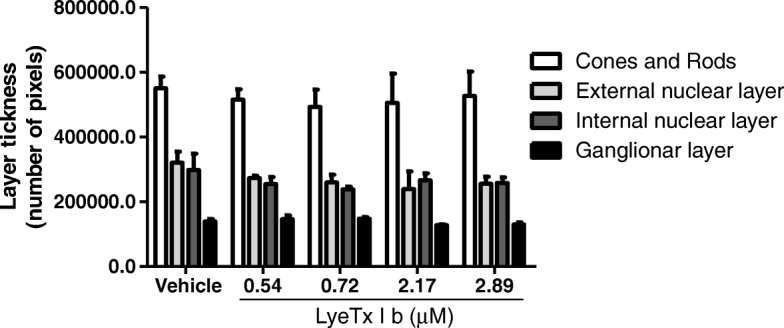
LyeTx I b does not alter the morphological integrity of the retina: Graph shows the measure of layer thickness of cones and rods, external, internal and ganglion layers 28 days after intravitreal injection of Vehicle, LyeTx I b 0.54 μM, LyeTx I b 0.72 μM, LyeTx I b 2.17 μM or LyeTx I b 2.89 μM. Data represent the means ± SEM of three independent experiments. No significant difference was observed as compared to vehicle group (p > 0.05). Abbreviations: SEM, standard error of the mean

Histological evaluation (Fig. 9) shows no alterations of retinal layers, indicating that LyeTx I b is nontoxic to the retina.

### LyeTx I b prevent neovascularization on CAM model

**Fig. 10:**
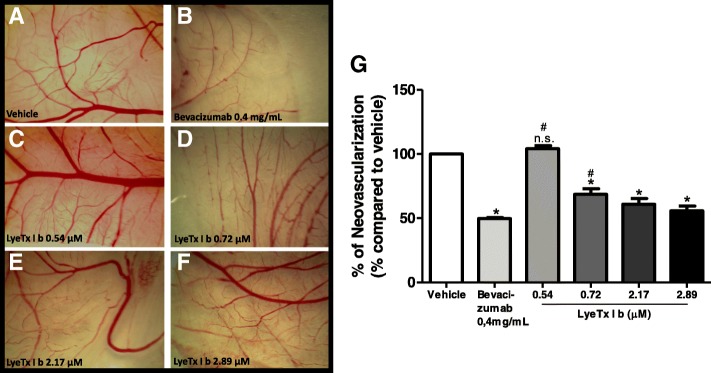
LyeTx I b prevents neovascularization on the CAM: Sequence of photographs illustrating the effect of (**a**) Vehicle, (**b**) Bevacizumab 0.5 mg/mL, (**c**) LyeTx I b 0.54 μM, (**d**) LyeTx I b 0.72 μM, (**e**) LyeTx I b 2.17 μM and (**f**) LyeTx I b 2.89 μM on the CAM over a 72-h period. **g** Graph shows the measure of vascularization after exposure to untreated eggs (Vehicle) or treatment with LyeTx I b (0.54 μM, 0.72 μM, b 2.17 μM or 2.89 μM). Data represent the ± SEM of number of pixels of twelve independent experiments. * indicates significant difference as compared to vehicle (*p* < 0.05). ^#^ indicates significant difference as compared to Bevacizumab (p < 0.05). n.s. indicates no significant difference as compared to vehicle (p > 0.05). Abbreviations: SEM, standard error of the mean

**Fig. 11:**
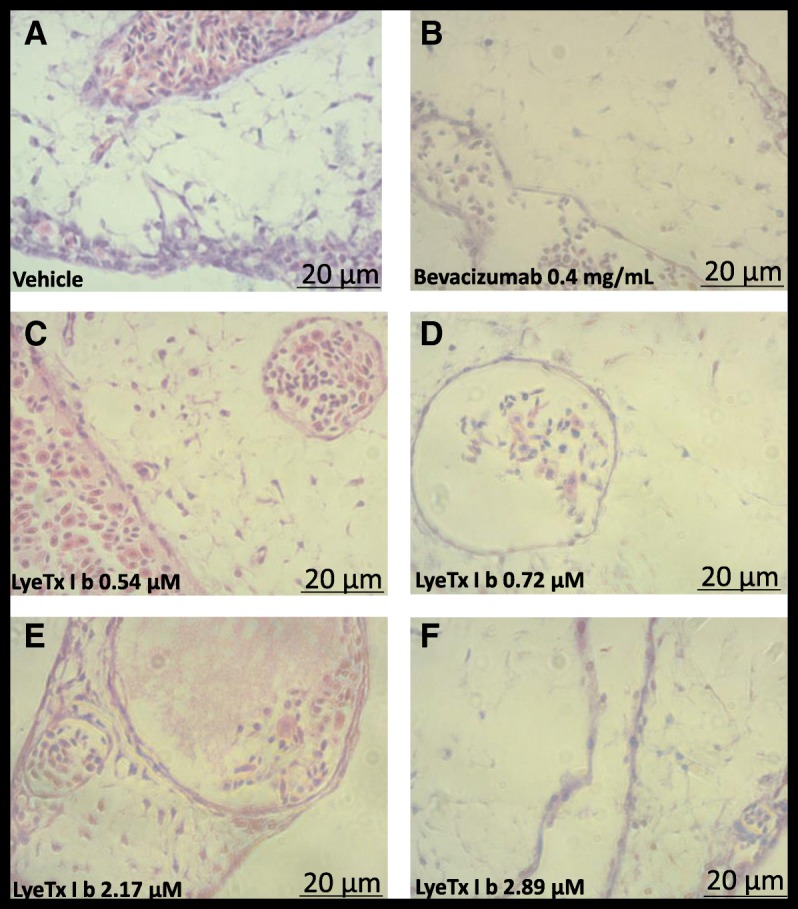
LyeTx I b prevents neovascularization keeping the morphology integrity of CAM: ***A:*** Histological images illustrating CAM mesoderm after exposure to (**a**) Vehicle, (**b**) Bevacizumab 0.5 mg/mL, (**c**) LyeTx I b 0.54 μM, (**d**) LyeTx I b 0.72 μM, (**e**) LyeTx I b 2.17 μM and (**f**) LyeTx I b 2.89 μM over a 72-h period. (**a** and **c**) Vasculogenic reaction: simultaneous and co-localized vasculogenesis and hematopoiesis is observed. **b**, **d**, **e** and **f** show reduction in vasculogenesis and hematopoiesis

Despite the absence of published studies on the role of the peptide in eye vascularization, it is important to investigate the behavior of LyeTx I b through models compatible with retinal vasculature. In this sense, CAM assays have been considered an appropriate model. Thus, we investigated primarily, in this traditional model of vascularization, whether CAM could present altered vascularization when exposed to LyeTx I b. Starting from the result that LyeTx I b did not affect viability of ARPE-19 cells, we tested its potential to reduce neovascularization. At the same concentrations used in animals the peptide was nontoxic in CAM at 0.54, 0.72, 2.17 and 2.89 μM (Fig. 10a, b, c, d, e and f, respectively). Interestingly, LyeTx I b at 0.54 μM promoted neovascularization at the same levels as the vehicle (Fig. 10g); however, the opposite effect was produced by the other concentrations, where LyeTx I b at 0.72 μM, 2.17 μM and 2.89 μM was able to prevent neovascularization (Fig. 10g). Importantly, LyeTx I b 2.89 μM did not alter the stromal layer of CAM at 0.54 μM, 0.72 μM, 2.17 μM or 2.89 μM (Fig. 11c, d, e, and f respectively) compared to vehicle (Fig. 11a), indicating the peptide was not toxic. Otherwise, the peptide was as effective at reducing neovascularization as Bevacizumab (0.4 mg/mL) (Fig. 11b) at a concentration one thousand times lower (Fig. 11f) preventing 50% of neovascularization without promoting toxicity to the embryo, thus indicating a safe LyeTx I b concentration for this purpose.

## Discussion

Retinal pigment epithelial (RPE) cells are central to retinal health and homoeostasis. RPE damage occurs as part of the pathogenesis of age-related macular degeneration and neovascular retinopathies [19]. In this study, we investigated the safety of different doses of LyeTx I b, a synthetic peptide derived from a toxin isolated from the venom of *L. erythrognatha*, in the rabbit vitreous, until 28 days after injection. Due to the absence of studies investigating the effects of this synthetic peptide on the eye, especially in retinal cells, we aimed to analyze the impact of LyeTx I b on the viability of ARPE-19 cells. LyeTx I b was active against different types of bacteria such as *E. coli*, whose minimum inhibitory concentration (MIC) was 0.71 μM, and against different types of fungal such as *Candida lusitanea* 11.52 μM of MIC [14] . To the best of our knowledge, the safety of intraocular injection of LyeTx I b has never been reported. Our study was designed to investigate possible toxic effects of LyeTx I b on the retina, taking into account that ARPE-19 cells did not exhibit apparent modifications on morphology when exposed to all concentrations of this peptide (Fig.1a), demonstrating the preservation of healthy culture. Furthermore, ARPE-19 cells treated with LyeTx I b presented the same viability levels as untreated ARPE-19. Importantly, the cell viability starts to decrease at 2.89 μM of the peptide, but those concentrations were able to keep cell viability above 50% (Fig.1b).

We evaluated the penetration efficiency of the FITC-labeled with LyeTx I b after intravitreal injection. It has been demonstrated that FITC is able to perfuse the retina [20]. In our study, retinal permeability, measured by the fluorescence of LyeTx I b –FITC, at 2 h, 4 h, 6 h and 8 h after injection (Fig. 2), demonstrated that intravitreal injection was successful and LyeTx I b penetrates progressively the retinal layers in this time range. (Fig. 2, b, c, d and e). These data are very important despite the absence of studies assessing the capacity of LyeTx I b - FITC to penetrate eye structures. In addition, we observed that even after 8 h of treatment this peptide did not spread to other layers of the retina, suggesting that LyeTx I b shows higher specificity for receptors in the RPE region.

Besides evaluating the efficiency of intravitreal injection, we examined the effects of LyeTx I b on ocular pressure at 7 days, 14 days and 28 days after intravitreal injection. Our results demonstrated no alterations on ocular pressure at 7, 14 or 28 days after treatment with the peptide, compared to vehicle (Fig.3a). In addition to that, we verified the safety of intravitreal injection of LyeTx I b 28 days after, comparing it to eyes before injection (Fig.3b). We did not observe alterations in intraocular pressure.

Furthermore, we decided to evaluate visual impairment of intravitreal through eletroretinography for 7, 14 or 28 days. At 28 days after the LyeTx I b intravitreal injection, no alterations were detected. The analysis of the b-wave amplitude variation according to the luminous stimulus intensity is a widely used method for the functional evaluation of the retina [21, 22].

Over the observation period the different doses of LyeTx I b did not affect overall retinal function. Nowadays, VEGF-inhibitors like bevacizumab, ranibizumab, pegaptanib, are the first choice in therapies for the treatment of neovascular ocular diseases. Some studies demonstrated transient changes in the electroretinograms after intravitreal injection of VEGF-inhibitors, although clinical adverse effects in the adult human eye are not common [23–26].

In different animal models, a combination of electrophysiological and histological examinations has been employed to evaluate drug safety. Rabbits present rod-dominated retina due to differences in retinal anatomy, which can explain why the ERG effects in rabbits are predominantly on the rod-mediated response [27]. Furthermore, we found evidence that LyeTx I b does not alter retinal function for long periods of treatment, except at 0.54 μM as indicated by (Fig. 4) where LyeTx I b promoted an increase of the wave times 14 and 28 days after, but interestingly, this finding is followed by the fact that cones and rods or ganglionic layers expression shows no alteration in the presence of LyeTx I b compared to vehicle (Fig. 9).

At the end of ERG responses of 7 days, 14 days and 28 days we evaluated the effects of LyeTx I b at 0.54 μM, 0.72 μM, 2.17 μM and 2.89 μM (Fig. 8 b, c, d, and e, respectively) in the eye fundus through Clear View® assembly to vehicle (Fig. 8a). Our findings indicate that LyeTx I b did not alter retinal vascularization at 7 or 14 days after (data not shown) whereas, importantly, 28 days after, LyeTx I b remains nontoxic to the retina assembly to vehicle.

It has been proposed that spider venoms are able to ameliorate retinal injury [28]. Despite the absence of previous reports about the effect of LyeTx I b on the eye, the results presented herein demonstrate that this peptide is biocompatible with the ocular system. Nevertheless, our data indicate that LyeTx I b is likely to be implicated in the decreased vascularization observed in CAM above 0.72 μM (Fig. 10g).

However, our data demonstrate for the first time that LyeTx I b is effective by itself at reducing neovascularization, at concentrations a thousand times lower than Bevacizumab, the treatment of reference, In addition, LyeTx I b did not promote inflammatory reaction in CAM (Fig. 10c, d, e and f). It is important to note that we examined the interaction between LyeTx I b and neovascularization in a native organism, without an installed disease. Besides that, LyeTx I b was administered in a single I.V. application, whereas Freitas et al (2013) [29] performed a clinical study using multiple intravitreal injections, during which all eyes developed cataract and one patient developed vitritis. One eye had mild persistent submacular fluid without active choroidal neovascularization, whereas the other eye had a persistent amount of intraretinal fluid due to active choroidal neovascularization. Our study of the synthetic peptide did not show the abovementioned alterations.

The treatment for retinopathies with bevacizumab appear to be good [30–33], but there are no studies demonstrating their safety when injected into the eye. Moreover, we demonstrate that LyeTx I b is a safe procedure useful for development of new studies focused on treatment of eye diseases that require I.V.I. for reduction of retinal vascularization.

In conclusion, the findings of this study strongly indicate that LyeTx I b can facilitate reduction of neovascularization with a single intravitreal injection and that, even 28 days post injection, no toxicity or morphological alterations of the retina were observed, up to a concentration of 2.89 μM. This finding suggests that the peptide is safe for intraocular injection. Therefore, additional studies need to be performed to verify the long-term safety of high doses of LyeTx I b in the retina. If this peptide proves to be safe, intraocular LyeTx I b might be considered as a possible new agent for the treatment of neovascularization in ocular diseases, such as macular edema, diabetic macular edema, and age-related macular degeneration.

## Abbreviations

AMD: Macular Age-Related Degeneration
CAM: Corio-allantoic membrane
ERG: Electroretinogram
FITC: Fluorescein Isothiocyanate
I.V.: Intravitreal
I.V.I.: Intravitreal injection
LyeTx I b: Synthetic peptide obtained from *Lycosa erithrognatha* spider venom
RPE: Retinal Pigment Epithelium
VEGF: Vascular Endothelial Growth Factor
